# Electrically Conductive Networks from Hybrids of Carbon Nanotubes and Graphene Created by Laser Radiation

**DOI:** 10.3390/nano11081875

**Published:** 2021-07-22

**Authors:** Alexander Yu. Gerasimenko, Artem V. Kuksin, Yury P. Shaman, Evgeny P. Kitsyuk, Yulia O. Fedorova, Artem V. Sysa, Alexander A. Pavlov, Olga E. Glukhova

**Affiliations:** 1 Institute of Biomedical Systems, National Research University of Electronic Technology MIET, Shokin Square 1, 124498 Moscow, Russia; nix007@mail.ru (A.V.K.); julia.laburnum@yandex.ru (Y.O.F.); 2 Institute for Bionic Technologies and Engineering, I.M. Sechenov First Moscow State Medical University, Bolshaya Pirogovskaya Street 2-4, 119991 Moscow, Russia; glukhovaoe@info.sgu.ru; 3 Scientific-Manufacturing Complex “Technological Centre”, Shokin Square 1, bld. 7 off. 7237, 124498 Moscow, Russia; shaman.yura@gmail.com (Y.P.S.); kitsyuk.e@gmail.com (E.P.K.); sysa.artem@yandex.ru (A.V.S.); 4 Institute of Nanotechnology of Microelectronics of the Russian Academy of Sciences, Leninsky Prospekt 32A, 119991 Moscow, Russia; pavlov.a@inme-ras.ru; 5 Department of Physics, Saratov State University, Astrakhanskaya Street 83, 410012 Saratov, Russia

**Keywords:** carbon nanotubes, graphene sheets, hybrid nanostructures, networks, laser radiation, electrical conductivity, hardness, nanoelectronics, bioelectronics, intelligent wearable devices

## Abstract

A technology for the formation of electrically conductive nanostructures from single-walled carbon nanotubes (SWCNT), multi-walled carbon nanotubes (MWCNT), and their hybrids with reduced graphene oxide (rGO) on Si substrate has been developed. Under the action of single pulses of laser irradiation, nanowelding of SWCNT and MWCNT nanotubes with graphene sheets was obtained. Dependences of electromagnetic wave absorption by films of short and long nanotubes with subnanometer and nanometer diameters on wavelength are calculated. It was determined from dependences that absorption maxima of various types of nanotubes are in the wavelength region of about 266 nm. It was found that contact between nanotube and graphene was formed in time up to 400 fs. Formation of networks of SWCNT/MWCNT and their hybrids with rGO at threshold energy densities of 0.3/0.5 J/cm^2^ is shown. With an increase in energy density above the threshold value, formation of amorphous carbon nanoinclusions on the surface of nanotubes was demonstrated. For all films, except the MWCNT film, an increase in defectiveness after laser irradiation was obtained, which is associated with appearance of C–C bonds with neighboring nanotubes or graphene sheets. CNTs played the role of bridges connecting graphene sheets. Laser-synthesized hybrid nanostructures demonstrated the highest hardness compared to pure nanotubes. Maximum hardness (52.7 GPa) was obtained for MWCNT/rGO topology. Regularity of an increase in electrical conductivity of nanostructures after laser irradiation has been established for films made of all nanomaterials. Hybrid structures of nanotubes and graphene sheets have the highest electrical conductivity compared to networks of pure nanotubes. Maximum electrical conductivity was obtained for MWCNT/rGO hybrid structure (~22.6 kS/m). Networks of nanotubes and CNT/rGO hybrids can be used to form strong electrically conductive interconnections in nanoelectronics, as well as to create components for flexible electronics and bioelectronics, including intelligent wearable devices (IWDs).

## 1. Introduction

Currently, the number of areas with the use of filamentous structures of carbon atoms—carbon nanotubes (CNT)—is growing rapidly. Carbon nanotubes are considered as promising functional additives to improve characteristics of piezoresistive elements [1,2], solar energy converters [3,4,5], and energy storage [6,7,8,9]. One of the main applications of CNT is creation of conductive channels with a high degree of conductivity [10,11], due to their excellent electrical, thermal and optical properties [12,13,14,15,16,17,18]. To create such channels, technologies for bonding nanotubes into electrically conductive networks with contacts that act as percolation nodes between CNTs are promising [19,20,21,22,23]. Graphene is no less promising a nanomaterial for electronics. Unique properties of graphene [24] and its derivatives [25] are the reason for their close study as the most promising materials for the creation of capacitors [26,27,28], aerogels [29,30] and sensors [31,32,33]. Among the problems encountered in the creation of graphene structures is the creation of structures with a low/high content of defects [34,35,36,37] and impurities [38,39,40,41], and the development of methods for defectiveness controlling. Methods of corrugation [42], deformation [43], as well as functionalization by atoms of various chemical elements [44,45] can be used to control the properties of graphene structures.

Significant advances have been made in the creation of hybrid materials that include CNTs and graphene/graphene oxide sheets due to the complementary structural and electrophysical features of two nanocarbon modifications. Using created hybrids of CNT and graphene sheets, such promising devices as intelligent wearable devices (IWDs) are being developed [46,47,48]. No less promising areas of CNT and graphene hybrids application are efficient batteries, supercapacitors, conducting channels, field emitter devices, and other electronic and optical devices. Properties of such CNT/graphene heterostructures have been studied in many works [49,50,51,52,53,54,55]. Hybrid-based composites have high electrical conductivity and are used as flexible strain gauges [46], heating elements [48], and flexible capacitors [56]. In supercapacitors based on CNT/graphene hybrid composites, condensing properties are provided by nanotubes. CNTs are also used to reduce the self-aggregation of graphene. During the charge-discharge process, they act as electrically conductive channels that facilitate the transport of electrons [57,58]. Improved conducting properties of CNT arrays grown on graphene layer are due to unsaturated π-bonds of edge CNT atoms, which lead to the formation of stronger bonds with atoms in graphene layer [59]. In the case of using CNT/graphene structures as a field emission device, stable bonds of CNT with graphene film are formed [60,61,62]. Due to high electrical conductivity of graphene and CNTs, electron transport and, as a consequence, uniform emission over the entire film area are ensured [63].

The prospects of using framework materials based on carbon nanoparticles increase when they are introduced into various media and matrices, which include metals, polymers, ceramics, and other materials [64,65,66,67,68]. However, it is important to understand the possibility of forming contacts between nanotubes and graphene sheets without matrix on a substrate [61,69,70]. By creating connections between CNT and other modifications of nanocarbon, it is possible to achieve a decrease in contact resistance and, as a consequence, an increase in electrical conductivity of the structures [71]. After synthesis, CNTs can be represented in the form of disordered systems and oriented arrays on a substrate, in various media and matrices. Currently, work is underway to synthesize connected nanotubes in the form of networks. For the synthesis of SWCNT networks, FCCVD gas-phase method [72] in H_2_ as a carrier gas is mainly used [73]. However, the methods of synthesizing nanotube networks are extremely difficult to control and difficult to implement. For this reason, methods of binding CNTs after synthesis by external influence are being actively developed. Such methods are based on the mechanisms of concentrated energy action, based on the latest achievements of quantum electronics, laser optics and precision mechanics.

Thus, there is a need to search for the methods of nanostructure formation from hybrids of carbon nanotubes and graphene. The cheapest and most universal approach for creation of such structures is the making of bonds between CNTs and nanoparticles under the influence of external electromagnetic fields [74]. Laser systems are widely available sources of electromagnetic radiation, which allow for non-contact and localized action on nanomaterials in order to change their structural and functional properties [63]. Modern lasers allow precise dosage of electromagnetic energy (power density in case of continuous radiation and energy density in case of pulsed mode) at given wavelengths [75]. Another advantage of using laser systems is their precision and automation when monitoring the parameters of laser irradiation in real time. It is possible to carry out structural modification of CNT by laser irradiation, which is accompanied by strong heating in confined space [76]. In this case, the versatility of non-contact high-speed irradiation of lasers has been demonstrated. The versatility lies in the ability to change the surface parameters of CNT films by setting the amount of energy per unit area by controlling the radiation energy density, frequency and number of pulses. In series of works, the effect of laser welding of MWCNTs was demonstrated using irradiation without the use of auxiliary substances, which are called solders [77,78,79,80]. A continuous wave Yb laser (λ = 1064 nm) and pulsed Nd:YAG laser (λ = 355 nm) were used to create connections between MWCNTs. Laser welding effect was achieved with both wavelengths at irradiation power density ~30 W/cm^2^ and irradiation time 3–6 s, as well as ~10 W/cm^2^ and 2–17 s. With an increase in Yb laser irradiation time, the resistance changed. It was shown that in the first 2 s resistance decreased from 118.4 to 3.8 kΩ, then it increased almost linearly to 92.2 kΩ. Most likely, the decrease in resistance was associated with a moderate degree of graphitization and formation of effective contacts between crossed nanotubes [81]. The physical mechanism of bond formation between nanotubes can be explained as follows. During the irradiation process, the energy is absorbed by electrons and then converted into the CNT atoms’ energy. Collision of phonons with carbon atoms leads to formation of defects, such as vacancies and interstices in the walls of nanotubes. Nanotubes also have a large number of defects after synthesis, as evidenced by the Raman spectroscopy of synthesized product. Ballistic collisions of electrons with carbon nuclei also lead to formation of defects [82]. Due to the moderate temperature, mobility of such defects leads to C–C bonds breaking in CNT walls [76]. New chemical bonds are formed on the contact surfaces of welded nanotubes. In the case of MWCNT, this leads to reconstruction of outer graphene layer surface and a decrease in the outer diameter of nanotubes. In the case of SWCNT, this process occurs on the lateral surface of the nanotube. Thus, it can be assumed that when graphene sheets are bonded to nanotubes, contacts will be formed between defect regions at graphene ends and lateral or end (open ends) defects of nanotubes.

In view of the foregoing, this work proposes a technology for formation of nanostructures from hybrids of single-walled (SWCNT), multi-walled carbon nanotubes (MWCNT) and reduced graphene oxide (rGO) on Si substrates. As a result of processing by scanning irradiation of nanosecond laser, the effect of nanowelding of CNT with graphene sheets was obtained. The formation process of covalent bonds between CNTs and graphene sheets under irradiation of laser beam has been simulated. For this, the theoretically most optimal laser wavelength, which provides the highest energy absorption, has been identified. Electrical conductivity of hybrid nanostructures exceeded electrical conductivity of nanotubes, since CNTs played the role of electrically conductive bridges. They contributed to formation of the most optimized structure with a large number of percolation sites. The superiority of obtained structures has been proven using molecular dynamics modeling and by comparing hardness and electrical conductivity of the samples. The networks from hybrids of CNTs and rGO created by laser radiation can be used to form strong electrically conductive interconnections in nanoelectronics, as well as to create components for flexible electronics and bioelectronics, including IWDs.

## 2. Materials and Methods

### 2.1. Method for Modeling of Contacts Formation between CNTs and Graphene Sheets

As mentioned earlier, binding of CNTs and graphene sheets can occur in defect areas due to the formation of covalent bonds, as well as due to the appearance of amorphous carbon as a solder in contact area [83]. In this case, bonding of MWCNTs with each other and with graphene occurs as in the case of SWCNT, only mainly in the outer walls region. We have previously proposed mechanisms for welding SWCNT together [68,84,85]. In these works, modeling of contact formation of various topologies between SWCNTs with different types of defects is presented. Mechanisms for the formation of suture and seamless X- and T-shaped contacts between SWCNTs have been identified. Moreover, the results of electronic transport simulation with calculation of resistance and electrical conductivity in such topologies of contacts between SWCNT and their bundles have been presented. Formation of covalent bonds is determined by amount of absorbed laser irradiation energy. The absorption coefficient is known to be a function of frequency. In this regard, an important point is to identify the most effective frequency range providing maximum absorption of incident electromagnetic wave. To calculate the absorption spectrum of nanotubes, a real-time time-dependent density functional tight-binding method is used [86]. It was implemented in DFTB + Version 20.2 software package [87]. The first step was to optimize the atomic structure using the self-consistent density functional tight-binding (SCC-DFTB) method, which allows you to determine system ground state [88]. Next, the effect of electromagnetic wave was simulated. To do this, we introduced an initial perturbation in the shape of a Dirac delta pulse. In this case, applied field was defined by expression:

(1) $$E\left(t\right)=E_{0}\delta \left(t-t_{0}\right)$$

where $E_{0}$—maximum value. Perturbed Hamiltonian is written as the sum of unperturbed and additional term representing the external perturbation:

(2) $$\hat{H}=\hat{H^{0}}+E_{0}\delta \left(t-t_{0}\right)\hat{\mu }$$

where: $\hat{\mu }$—dipole moment, $\hat{H^{0}}$—unperturbed Hamiltonian, defining ground state, constructed using applied SCC-DFTB method. The electric field strength was taken to be equal to 0.001 V/Å. After electromagnetic wave action, density matrix $\hat{\rho }$ evolves. For its calculation the Liouville–von Neumann equation was integrated:

(3) $$\frac{\partial \hat{\rho }}{\partial t}=\frac{1}{i\hbar }\left[\hat{H},\hat{\rho }\right]$$

As is known, if the momentum of external field is relatively small, then the response of system under consideration is linear and the dipole moment was determined in this case as follows:

(4) $$\mu \left(t\right)=\int_{-\infty }^{\infty }\alpha \left(t-\tau \right)E\left(\tau \right)d\tau ,$$

where α(t−τ)—polarizability along the axis over which the external field E(t) is applied. Amount of external field energy absorption is determined by imaginary part of polarization coefficient, which is obtained as a result of Fourier transform of time-dependent dipole moment and can then be in the following form:

(5) $$\alpha \left(\omega \right)=\frac{\mu \left(\omega \right)}{E_{0}}.$$

As a result, frequencies with maximum energy absorption are determined from absorption spectrum.

Further, the Ehrenfest TD-DFTB method [86] was applied, which allows us to correctly investigate the dynamics of nuclei and electrons under laser irradiation. Laser irradiation frequency was taken to be equal to that which was determined at the previous stage of the study. As a result, regularities of charge flow between welded objects, in our case, between tubes and graphene sheets, are revealed.

### 2.2. Method of Creating Homogeneous Dispersed Media from Carbon Nanomaterials

Initially, homogeneous dispersed media based on carbon nanomaterials SWCNT, MWCNT, and rGO were prepared. We used carbon nanomaterials that are synthesized by the most accessible and popular methods. SWCNT (OCSiAl Ltd., Moscow, Russia) were obtained by gas-phase synthesis SWCNT. The average diameter was 1–2.5 nm, their length was about 5 µm, and the specific surface area was 420 m^2^/g. Using the analysis of Raman spectra [89,90,91], the chirality indexes (x,y) were determined for SWCNT: (8,4); (16,0); (10,6); (19,17); (19,8); (15,8); (15,5); (13,6). MWCNT (NanoTechCenter Ltd. (Taunit), Tambov, Russia) were synthesized by gas-phase chemical deposition. The average diameter was 15–40 nm, their length was about 2 µm, and the specific surface area was 120 m^2^/g. rGO was prepared by modified Hammers method. For this, 1 g of graphite powder was mixed with 6 g of potassium permanganate (KMnO_4_). A total of 14 mL of phosphoric acid (H_3_PO_4_, 85%) was added to the mixture. Then, the whole mixture was mixed with 120 mL of concentrated sulfuric acid (H_2_SO_4_, 95%). The resulting suspension was placed in a drying oven, where it remained at a temperature of 50–60 °C for 12 h. After heat treatment, the resulting suspensions were mixed with water in an amount of 140 mL. Hydrogen peroxide (H_2_O_2_, concentration 30%) was added to the resulting aqueous mixture until foaming ceased (~3 mL). Then, sediment was separated from the resulting dispersion using centrifuge at 12,000 g for 30 min. The resulting material was washed with water using centrifuge to pH 5. Reduction of graphene oxide was carried out in two stages: annealing in muffle furnace at temperature 200 °C for an hour; annealing in argon and hydrogen at 1000 °C with volume ratio 1:1 for 1 h. The number of rGO graphene layers did not exceed 4. RGO contained C–H bonds in its structure [92].

A total of 4 dispersed media with different compositions were prepared (Table 1). Preparation was carried out by mixing carbon materials with deionized water and solvent. Bio-surfactant sodium cholate hydrate (SC) was used as a solvent to create dispersed media. Ratio of SC to SWCNT was 1:4 by weight, ratio of SC to MWCNT was 1:2 by weight, SC to rGO was 1:2 by weight. Dispersions’ compositions were as follows: dispersion 1—SWCNT 0.1 mg/mL, dispersion 2—MWCNT 0.1 mg/mL, dispersion 3—SWCNT/rGO 0.05/0.05 mg/mL, dispersion 4—MWCNT/rGO 0.05/0.05 mg/mL.

After mixing, the dispersed media were treated with Q700 Sonicator submersible sonicator (Qsonica Ltd., Newtown, CT, USA) for 10 min with power 150 W/cm^2^. Next, the dispersions were sonicated in an Elmasonic S30H bath (Elma Ltd., Singen, Germany) with power 80 W for 60 min.

### 2.3. Method of Thin Layers Application

**Figure 1:**
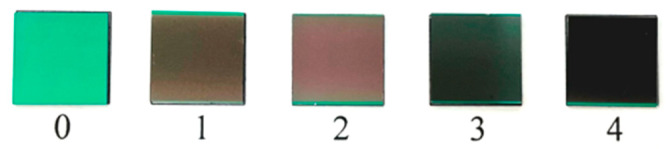
Thin films sputtered on oxidized Si substrates: (**1**) SWCNT, (**2**) MWCNT, (**3**) SWCNT/rGO, (**4**) MWCNT/rGO compared to (**0**) pure oxidized Si.

To form nanomaterial films, the dispersed media were deposited layer by layer on substrates. Si wafers with an oxide layer thickness of 0.52 μm and size 10 mm × 10 mm were chosen. The thickness of silicon oxide layer determines the observed color of 0 sample (Figure 1). In our case, green color is equivalent to thickness of silicon oxide 0.5–0.55 µm [93]. Substrates provided low thermal conductivity with carbon nanomaterials [78]. The substrates were preliminarily cleaned in an ultrasonic bath. Spray deposition method was used for deposition. For this, modified E2V dispensing systems installation (Nordson EFD, Westlake, OH, USA) was used. Pressure for feeding the dispersed medium through pneumatic nozzle was 2 bar. Nozzle diameter was 0.5 mm. The substrates were placed on a heating stage for instant evaporation of solvent from media. The stage was heated to a temperature of 120 °C. To form films ~500 ± 100 nm thick, 800–1000 layers were deposited, depending on the type of carbon nanomaterial.

Thus, four groups of samples were obtained for research, which were oxidized Si substrates with films made of SWCNT (1), MWCNT (2), SWCNT/rGO (3), MWCNT/rGO (4) (Figure 1). Each group contained 15 samples to obtain statistical results during research. Pure oxidized Si substrate is shown for comparison (0) (Figure 1).

### 2.4. Method of Laser Formation of Carbon Nanomaterials Films

Laser setup was used to create nanostructures from SWCNT, MWCNT, rGO and their hybrids on substrates. The main device of setup was an Nd:YAG pulsed laser, generating radiation at the fourth harmonic with wavelength in UV range 266 nm. The laser operated in pulsed mode with pulse duration 100 ns and frequency 30 kHz. It is known that pulsed radiation of short duration generates nonlinear optical effects in carbon nanotubes and graphene [75,94]. The laser setup had a galvanometric scanning system. Irradiation pattern of deposited films was a square consisting of individual laser pulses with spot diameter ~35 μm. Distance between the spots centers was 17 μm. Beam speed was 500 mm/s. Laser beam profile was Gaussian. Energy density of laser radiation was in the range 0.14–0.8 J/cm^2^. Creation of nanostructures took place in Ar inert gas.

### 2.5. Materials Characterization

#### 2.5.1. Scanning Electron Microscopy

The study of topological features of hybrid nanostructures of carbon nanotubes and graphene sheets, which were films on Si substrates, was carried out using scanning electron microscope FEI Helios NanoLab 650 (FEI Ltd., Hillsboro, OR, USA). Accelerating voltage of electron column was 5 kV, electron probe current was 50 pA. Pressure in the vacuum chamber was 3.9 × 10^−4^ Pa.

#### 2.5.2. Raman Spectroscopy

It is known that Raman spectroscopy is an effective tool for controlling the structure of carbon nanomaterials (nanotubes and graphene) [95]. The efficiency of using this method for studying the structural features of carbon nanomaterials after laser exposure was demonstrated in works [63,78,96]. Therefore, in this work, control of the ratio I_D_/I_G_, broadening of peaks and changes in their intensity in Raman spectra was carried out.

Raman spectra of film samples were obtained in the backscattering geometry on LabRAM HR Evolution (Horiba Ltd., Villeneuve-d’Ascq, France). Ar laser (514 nm, power during the recording of spectra 0.125 mW) was used as a source of exciting radiation. The diffraction grating of 1800 grades/mm provided spectral resolution 0.5 cm^−1^. CCD camera was cooled to 205 K to record Raman scattering on the Stokes side in a wide spectral range. Precision motorized stage and an BX41 (Olympus Corp., Tokyo, Japan) built-in microscope were used to focus the laser beam on the study area. The signal accumulation time was 15 s averaging over 3 spectra to improve signal-to-noise ratio.

#### 2.5.3. Hardness Measurement

Hardness is directly related to the strength of nanostructures [96]. Therefore, to carry out hardness measurements of samples, a NanoScan-4D Compact nanohardness tester (TISNUM Ltd., Moscow, Russia) with indentifying tip of Berkovich pyramid shape was used. Elastic modulus of samples was measured by smoothly immersing indenter to 200 nm depth. Distance between the measurement points was 100 µm. Time to reach the required depth (Load time) was 10 s, and holding time of the achieved load (Hold time) was 1 s.

#### 2.5.4. Conductivity Measurement

Electrical conductivity of carbon nanomaterials films was determined using four-probe method. Four probes of PM5 station (Cascade Microtech Ltd., Beaverton, OR, USA) were located at the edges of sample composite layer. Probe station contacts were connected to 34401A multimeter (Keysight Technologies Ltd., Santa Rosa, CA, USA). Initially, resistance of the samples was measured (at least 5 times), then average resistance value was calculated.

## 3. Results and Discussion

### 3.1. Modeling the Process of Forming SWCNTs and Graphene Sheets Hybrids

**Figure 2:**
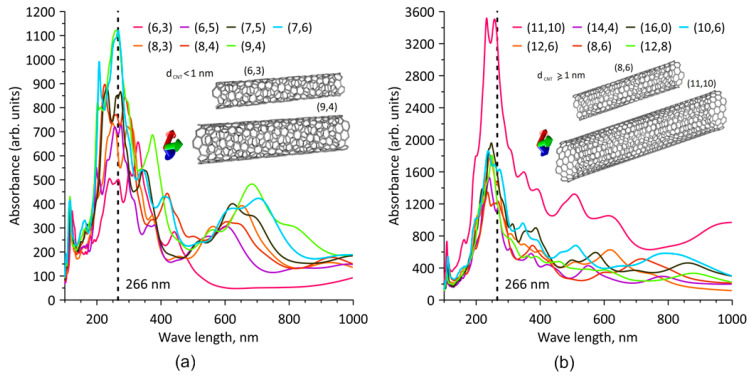
Absorption spectra for nanotubes of (**a**) subnanometer diameter and (**b**) diameters of 1 nm and more.

Tubes of different chiralities, the most frequently synthesized ones, were selected for the study. As is known, most of synthesized tubes are semiconductors. It is also known that the least amount of non-chiral tubes is synthesized from the total number. Therefore, for the study, we chose nanotubes of following types: (6,5), (6,3), (7,5), (12,6), (8,3), (8,4), (7,6), (12,8), (14,4), (10,6), (8,6), (9,4), (11,10) and (16,0) [97]. Series of calculations were carried out for indicated tubes of the same length of ~4–5 nm. To ensure that length of nanotubes was approximately the same, a corresponding different number of unit cells was taken for different nanotubes. Investigations were also carried out for the same nanotubes with 2–10 times greater length. Figure 2 shows absorption plots for all the listed nanotubes of 4–5 nm length. For thin tubes of subnanometer diameter, absorption curves are shown in Figure 2a; for tubes with diameter 1 nm and more, curves are shown in Figure 2b. All tubes show the same tendency towards maximum absorption in the UV region. In particular, in the wavelength range 200–300 nm, the largest number of absorption peaks is observed, regardless of chirality type of nanotube. Particularly prominent are wavelengths in the region of 266 nm. This region contains intensity peaks for the largest amount of SWCNTs of various chiralities. In this case, the most pronounced peaks at this wavelength are observed in semiconductor tubes. The intensity peak for the tube (11,10) is especially noticeable, which is explained not only by semiconductor type of conductivity, but also by the largest diameter of this particular tube in comparison with others, at 1.45 Å. The larger the surface, the more energy it will absorb from incident electromagnetic wave. Fermi energy for different types of tubes is 5.1 ± 0.3 eV. As noted above, tubes of different lengths were considered, and it was found that an increase in tube length did not qualitatively affect the absorption spectrum. That is, short and long SWCNTs have the largest peak absorption intensity in the wavelength region of about ~266 nm. Experimental absorption spectra for SWCNT (OCSiAl Ltd.) were given in [98], in Figure 1. Absorption spectra of MWCNT (NanoTechCenter Ltd. (Taunit)) were given in [99], in Figure 1. It can be seen from the absorption spectra that absorption decreases when going from UV to visible and IR ranges. In visible and near-IR ranges, there are also sloping regions of increase in absorption, the amplitude of which is not comparable with amplitude in UV region.

**Figure 3:**
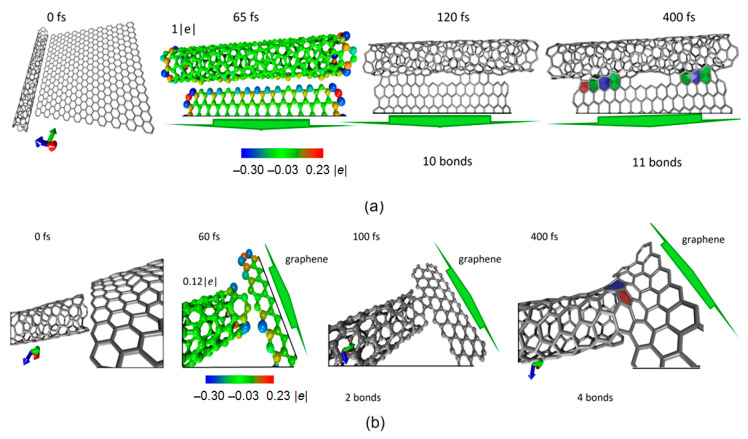
Nanowelding of a nanotube (6,3) and graphene sheet at different positions of graphene sheet: (**a**) graphene edge is located along the nanotube, (**b**) graphene edge is located end-to-end with nanotube edge.

**Figure 4:**
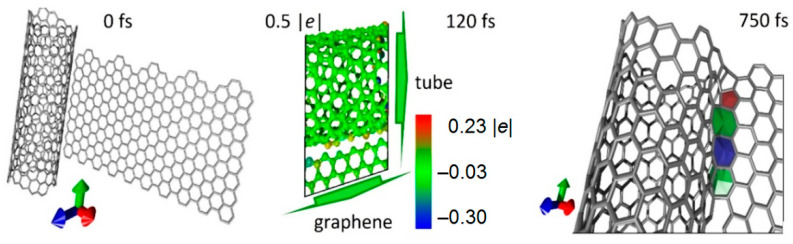
Nanowelding of nanotube (14,4) and graphene sheet at different positions of graphene sheet.

Next, we simulated the interaction of nanotubes with graphene sheets under laser irradiation with a wavelength of 266 nm. Previously, authors carried out series of numerical experiments, which resulted in formation of covalent bonds between nanotubes. It was shown that covalent bonds are formed primarily in the regions of defects in nanotubes. In this paper, we investigate nanowelding of defect-free tubes with graphene sheets. Various mutual positions of nanotube and sheet are considered. In all cases, graphene sheet was located at a distance of 2.8–3.0 Å. The results are presented in Figure 3 for thin tube (6,3), with diameter 0.6 nm, and in Figure 4 for tube (14,4), with diameter 1.3 nm. Figure 3a shows an atomistic model of nanotube and graphene sheet when graphene edge is located along the tube. The results of modeling and the process of covalent bond formation are shown. Under laser irradiation, redistribution of electron charge density occurs. As a result, charge transfer occurs: from nanotube, one electron is transferred to graphene. Figure 3a shows that the edge graphene atoms have an excess charge (blue), and nanotube atoms in the immediate vicinity of graphene have a lack of charge (yellow). As early as 120 fs after the onset of irradiation, first covalent bonds appear; after 400 fs, contact between nanotube and graphene is completely formed. The same figure shows non-hexagonal elements in nanowelding region. Pentagon is marked in red, heptagons in blue and octagons in green. Figure 3b also shows the process of nanotube–graphene contact formation when graphene sheet is placed not along the nanotube, but end-to-end with it. In this case, bonds are formed faster, and the first covalent bonds are formed already after 100 fs. As in the previous version, already in first moments, a redistribution of charge is observed and charge transfer from nanotube to graphene occurs again, although to a lesser degree in this case. As a result, four stable bonds were formed in 400 fs, and heptagon, pentagon, and hexagon appear in the contact region. In all cases, bond length in contact region is ~1.54–1.57 Å, which corresponds to C–C bond length in the case of sp3 hybridization of electron clouds.

Similar results were obtained for a nanotube with diameter twice as large. From the data presented in Figure 4, it can be seen that atoms of nanotube located next to graphene have a lack of charge. It can be said that these atoms and graphene atoms interact electrostatically. The nanotube loses its charge again, as in the case of the thin tube (6,3). However, unlike the thin nanotube, bonds are formed more slowly. Stable bonds appear not in 400 fs, but in 750 fs. Non-hexagonal elements are also formed in contact area.

### 3.2. Structural Features of the Created Carbon Nanomaterials

**Figure 5:**
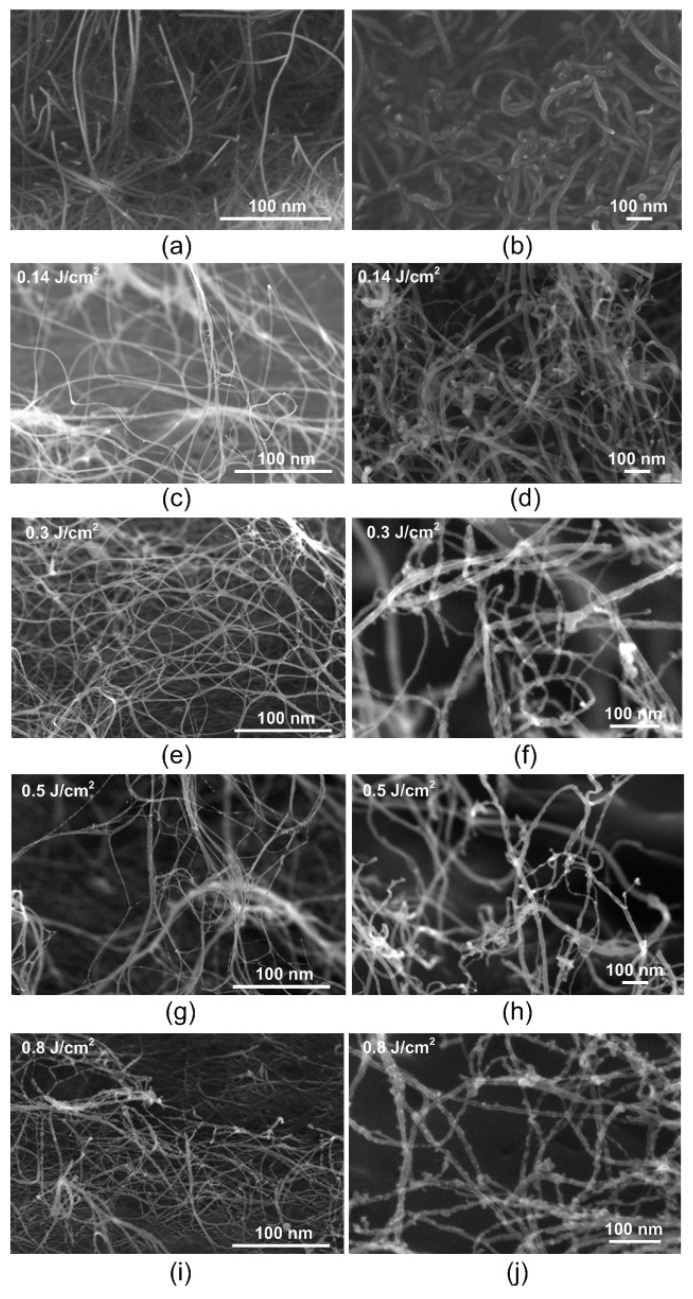
SEM images of (**a**,**c**,**e**,**g**,**i**) SWCNT and (**b**,**d**,**f**,**h**,**j**) MWCNT samples (**a**,**b**) before and (**c**–**j**) after laser irradiation with different energy densities: (**c**,**d**) 0.14 J/cm^2^, (**e**,**f**) 0.3 J/cm^2^, (**g**,**h**) 0.5 J/cm^2^, (**i**,**j**) 0.8 J/cm^2^.

Structure change of films of SWCNT and MWCNT networks after laser irradiation compared to initial CNT films is shown in Figure 5. SEM images show that SWCNT and MWCNT are presented as isolated nanotubes and their bundles. This is especially pronounced for SWCNT (Figure 5a,c,e,g,i). Initially, the energy density of laser radiation was determined, which, on the one hand, ensures formation of nanotube networks due to laser nanowelding, and, on the other hand, does not initiate the process of carbon nanostructure sublimation. SEM images of SWCNT films show that after laser irradiation with energy density 0.14 J/cm^2^ (Figure 5c), networks began to form compared to initial nanotubes (Figure 5a). Laser irradiation with energy density 0.3 J/cm^2^ led to formation of a branched SWCNT network. Laser energy is converted into the energy of atoms and promotes breaking of C–C bonds inside nanotubes with further formation of new bonds between atoms of neighboring CNTs. Laser energy density 0.5 J/cm^2^ (Figure 5g) and especially 0.8 J/cm^2^ (Figure 5i) ensured structural disruption. Such structural disturbances, most likely, are not associated with formation of vacancy defects, but are more similar to the appearance of condensation products—nanosized inclusions on the surface of nanotubes. Thus, appearance of such nanoinclusions can be associated with sublimation of defective nanotubes with subsequent condensation of amorphous carbon on nanotubes that have not been irradiated by laser [77,79].

A similar situation developed for MWCNT film, but threshold radiation energy density was higher than for SWCNT. The MWCNT network with the highest number of bonds between nanotubes was obtained at the energy density of 0.5 J/cm^2^ (Figure 5h). It can be seen from SEM images that with an increase of energy density from 0.3 to 0.5 J/cm^2^, MWCNTs were bent more due to change in defectiveness [80]. This contributed to formation of more bonds between nanotubes. At 0.81 J/cm^2^, the appearance of nanoinclusions was observed on MWCNT as on SWCNT. Conclusions about the optimal energy density for the formation of carbon nanotube networks cannot be made only on the basis of SEM images. The main quality criteria for the formed networks of nanomaterials are an increase in values of their electrical conductivity and hardness. However, based on SEM images, it is possible to determine the value of threshold irradiation energy density at which defect regions of nanotubes do not sublimate with their subsequent condensation, as in Figure 5g,i,j. As a result, energy density values 0.3 and 0.5 J/cm^2^ were chosen to irradiate SWCNT/rGO and MWCNT/rGO hybrids films, respectively.

**Figure 6:**
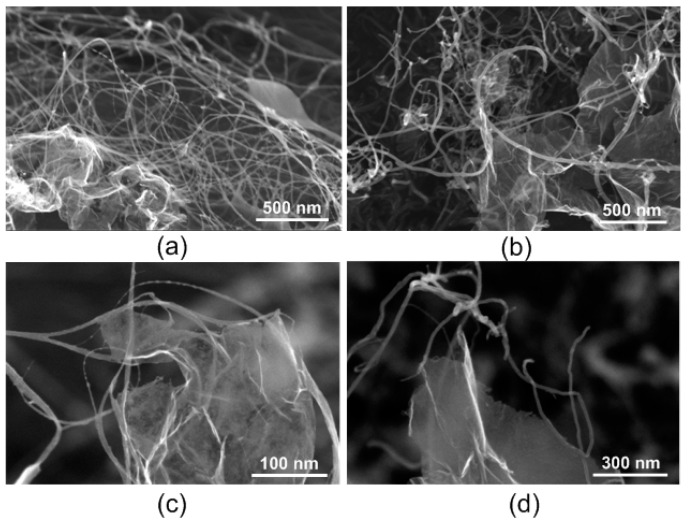
SEM images with different magnification of hybrid structures (**a**,**c**) SWCNT/rGO and (**b**,**d**) MWCNT/rGO after laser irradiation.

When exposed to laser irradiation on SWCNT/rGO and MWCNT/rGO films with selected values of energy density, the effect of CNT binding to rGO sheets was obtained (Figure 6). It can be seen on SEM images that binding of SWCNTs and MWCNTs, as well as their bundles with rGO, occurred mainly in regions of defects on nanotubes’ lateral surface with graphene sheet’s edge region (Figure 6c,d). Nanotubes also bonded to each other. Figure 6a,b show that nanotubes and their bundles acted as connecting bridges between graphene sheets.

**Figure 7:**
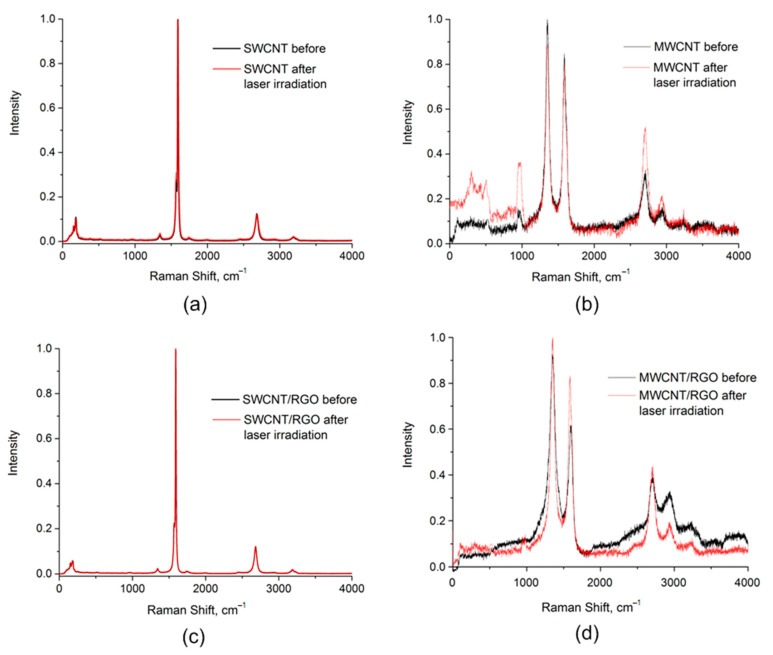
Raman spectra of (**a**,**b**) CNT and (**c**,**d**) CNT/rGO hybrids before (black line) and after (red line) laser irradiation with energy density (**a**,**c**) 0.3 J/cm^2^ for SWCNT and SWCNT/rGO, (**b**,**d**) 0.5 J/cm^2^ for MWCNT and MWCNT/rGO.

Figure 7 shows the Raman spectra of SWCNT and MWCNT (Figure 7a,b) samples and SWCNT/RGO and MWCNT/RGO hybrids (Figure 7c,d) before and after exposure to laser radiation with selected energy densities. The presented graphs reproduce the main characteristic modes of the samples: RBM (0–300 cm^−1^), D (1300–1400 cm^−1^), G (~1580 cm^−1^) and 2D (2500–2900 cm^−1^) for SWCNT, D (1300–1400 cm^−1^), G (1580–1600 cm^−1^) and 2D (~2700 cm^−1^) for MWCNT. RBM mode is typical for SWCNT samples. It is characteristic of the nanotube cylindrical geometry and is caused by uniform radial displacement of atoms. In this regard, it slightly depends on the atomic structure and is insensitive to small deviations of the nanotube surface from the ideal cylinder. The D mode originates from a double resonance Raman scattering process [95]. The D mode is observed for the presence of defects in the graphite structure and its intensity is proportional to the amount of disorder (crystallite boundary) in the sample [100]. It is known that the G mode, corresponding to the in-plane optical phonon modes, is characteristic of all sp2 carbon materials, and the defectiveness of the carbon structure is estimated by the I_D_/I_G_ intensity ratio.

The values of the characteristic modes for each sample are presented in Table 2. The data in the table were obtained by processing Raman spectra. Table 2 shows the frequency values for the main modes that characterize carbon nanomaterials (RBM, G, D, 2D), as well as the ratio of D and G modes’ intensities—I_D_/I_G_. For the initial SWCNT, a small value of the I_D_/I_G_ ratio characterizes a relatively low defectiveness. Films from SWCNT networks after laser irradiation with an energy density of 0.3 J/cm^2^ received an increase in the I_D_/I_G_ parameter by 0.008 (28%), which corresponds to an increase in the defectiveness of the nanostructure. Defectiveness of SWCNT nanostructures is mainly associated with the formation of vacancy defects with the breaking of C–C bonds and the formation of new bonds on the lateral surface of nanotubes. Along with an increase in defectiveness, broadening of the 2D mode is observed. The low-frequency mode G^–^ does not disappear with an increase in the intensity of mode D. This characterizes the appearance of defects in the structure that are not directly caused by graphitization of the sample. Such defects are probably caused by the formation of joints at the sites of defects on the nanotubes’ walls, which suggest a distortion of the original symmetric carbon structure. After exposure to the laser, SWCNTs did not undergo significant damage, which is confirmed by the preservation of the spectrum shape and the absence of additional high-frequency modes, such as D′ band intensity (around 1620 cm^−1^), which is often observed in defective graphene samples [100].

The ratio of the I_D_/I_G_ intensities of the initial MWCNT is greater than 1, which characterizes a higher defect rate in comparison with SWCNT. The presence of bands of the initial MWCNT in the spectral range from 100 to 1000 cm^−1^ refers to the contribution from the catalyst remaining after synthesis. For the initial MWCNT and MWCNT after laser exposure, a decrease in I_D_/I_G_ by 0.095 was obtained, which is associated with partial annealing of amorphous carbon from the surface of the outer walls of the nanotubes. This is confirmed by an increase in the intensity of the 2D mode. Moreover, after laser exposure, MWCNT is characterized by an increase in the D + G mode of about 2940 cm^−1^, which is a defect-induced two-phonon process. SEM images confirm that the defectiveness for MWCNT after laser exposure on one side is reduced by annealing the surface of the outer walls of the MWCNT. On the other side, the spectrum characterizes the presence of various defect types in the structure associated with the appearance of curvatures and the formation of bonds between CNTs at the sites of defects.

The spectra of SWCNT/rGO hybrids after exposure to laser radiation in comparison with the same without laser irradiation demonstrate a number of changes. First, the number of maxima in the region of the RBM mode has decreased. This can be explained by the fact that tubes of smaller diameter have less heat capacity and are much less resistant to heating from laser radiation, as a result of which they are destroyed faster. In addition, the heating of SWCNT/rGO hybrids is not uniform, and the presence of defects additionally creates uneven heating areas. Thus, as a result, larger diameter tubes prevail in the sample [101]. Secondly, the value of the I_D_/I_G_ parameter has sharply increased by 4.4 times. The shift of the G mode higher in frequency characterizes the change in the length of C=C bonds, probably due to the emergence of new C–C bonds between the tubes and rGO [102,103,104].

The MWCNT/rGO spectrum after laser irradiation with an energy density of 0.5 J/cm^2^ is characterized by a significant shift in the G mode, broadening of the D mode, and an increase in the I_D_/I_G_ ratio by 0.031 (~3%), which may be ascribed to the increment in sp2 domains caused by attaching carbon nanotubes to rGO; it could also be associated with decreasing of the outer diameter of nanotubes. This is consistent with a change in the shape of the spectra in the 2000–4000 cm^−1^ region, in particular, with changes in the 2D and D + G modes. It is also worth noting that the SWCNT and SWCNT/rGO samples are characterized by resistance to laser radiation with an energy density of 0.3 J/cm^2^, and the MWCNT and MWCNT/rGO samples with a value of 0.5 J/cm^2^, since the shape of the spectra was preserved relative to the untreated samples.

Presented data demonstrate that laser irradiation with certain energy density leads to formation of defects (change in I_D_/I_G_) in the structure of nanotubes for all samples without amorphization of the structure. The change in I_D_/I_G_ is mainly due to the welding of carbon nanotubes to each other and to graphene sheets. In this regard, it can be assumed that electrical conductivity of the obtained networks of nanotubes can slightly decrease due to the appearance of vacancy defects and can greatly increase due to the appearance of new contacts between nanomaterials.

### 3.3. Hardness of Nanomaterials

**Figure 8:**
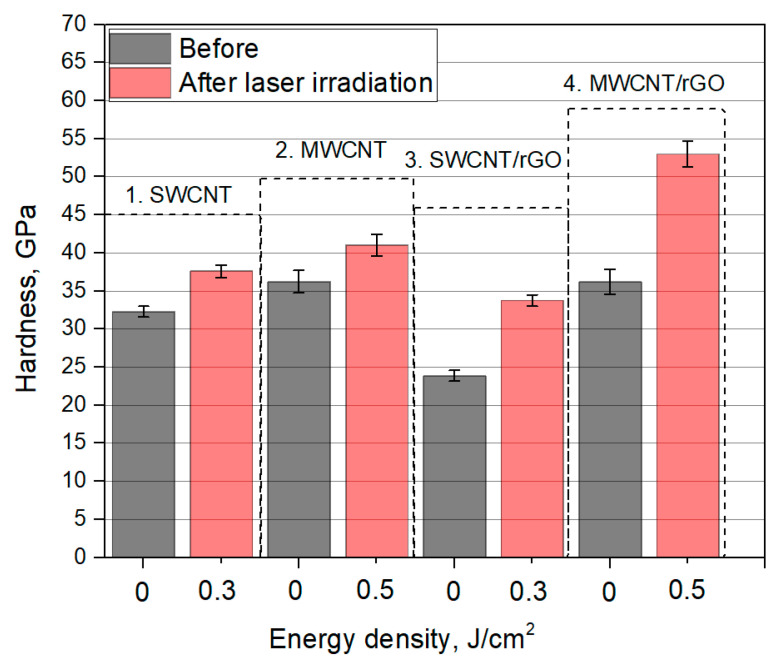
Hardness of films before (black columns) and after (red columns) laser irradiation, with energy density 0.3 J/cm^2^ for SWCNT and SWCNT/rGO, 0.5 J/cm^2^ for MWCNT and MWCNT/rGO.

To determine quantity and quality of joints between nanotubes, as well as nanotubes and graphene sheets, it is necessary to compare hardness of obtained films from nanomaterials. For each sample, five measurements were carried out. Average hardness values were calculated. Based on the data obtained, a graph of films’ hardness before and after laser irradiation was plotted (Figure 8).

SWCNT and MWCNT films before laser irradiation had a hardness of 32 GPa and 36 GPa, respectively. As a result of laser irradiation, hardness increased to 38 GPa and 41 GPa. This corresponds to an increase of 1.2 times and by 1.1 times, respectively. Laser irradiation of SWCNT/rGO and MWCNT/rGO hybrids results in a more significant increase in hardness compared to initial films. For SWCNT/rGO, an increase in hardness of 1.4 times is obvious, for SWCNT/rGO of 1.5 times. MWCNT/rGO film had the highest hardness of 54.5 GPa. Such a change in mechanical characteristics of the films confirms the data, obtained by Raman and SEM studies. The most mechanically strong networks were obtained on the basis of MWCNT and rGO hybrids. In this case, C–C bonds were formed not only between MWCNTs, but also between MWCNTs and rGO. It was found that films containing MWCNT are less stable, since a larger spread in hardness values was obtained for the same samples from the batch in comparison with samples based on SWCNT.

### 3.4. Electrical Conductivity of Nanomaterials

When quantity and quality of connections between nanotubes changes, as well as between nanotubes and graphene sheets, their electrical conductivity changes. Therefore, electrical conductivity of films based on investigated carbon nanomaterials was calculated. Surface resistance of films made of SWCNT, MWCNT, and their hybrids with rGO was measured before and after laser irradiation with energy densities of 0.14 J/cm^2^, 0.3 J/cm^2^, 0.5 J/cm^2^, 0.8 J/cm^2^. Then, based on the values of surface resistance, specific electrical conductivity of films was calculated taking into account their thickness. Electrical conductivity measurement results are shown in Table 3.

Thickness of the films made of carbon nanomaterials was ~500 ± 100 nm. Laser had a diffraction length (Rayleigh length) of ~1 mm. Thus, laser irradiation efficiency on the film had the same energy density throughout the entire thickness of the film. Therefore, it can be assumed that electrical conductivity of the formed films from nanotubes and their hybrids with rGO are the same on the surface and inside the films.

**Figure 9:**
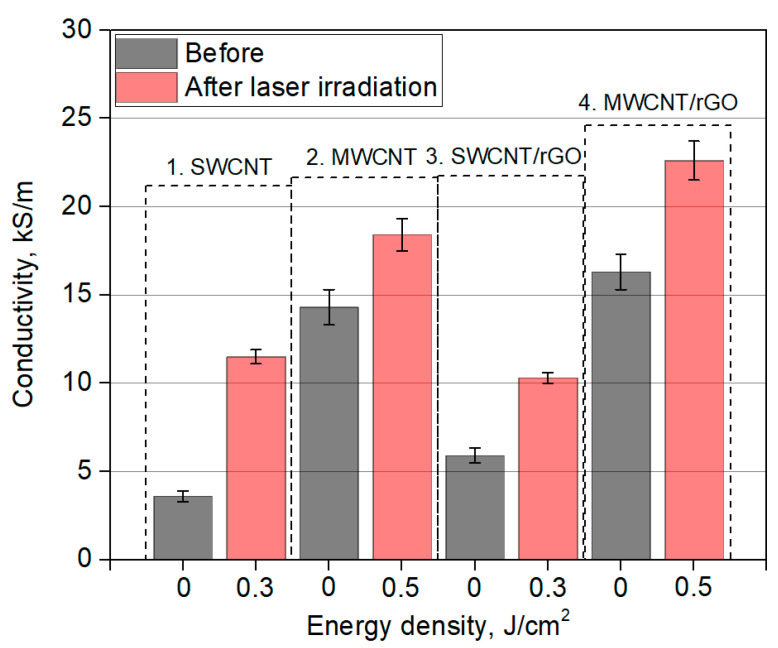
Electrical conductivity of films before (black columns) and after (red columns) laser irradiation with energy density 0.3 J/cm^2^ for SWCNT and SWCNT/rGO, 0.5 J/cm^2^ for MWCNT and MWCNT/rGO.

According to electrical conductivity measurement results, it can be seen that for all samples there is dependence of electrical conductivity on the laser energy density. Irradiation with energy density 0.14 J/cm^2^ led to an insignificant increase in electrical conductivity of films based on SWCNT and MWCNT. This result is in good agreement with obtained SEM images. Laser irradiation initiates formation of contacts between nanotubes; this helps to improve transport of electrons. Further increase of energy density to 0.3 J/cm^2^ leads to an even greater increase in conductivity values for films based on SWCNT and MWCNT networks, because even more connections between CNTs are being formed. However, irradiation of SWCNT films with energy density 0.5 J/cm^2^ leads to a decrease in electrical conductivity; irradiation of MWCNT films leads to an increase in electrical conductivity. Further increase in energy density to 0.8 J/cm^2^ in both samples causes structural destruction of nanotubes and, as a consequence, deterioration of conductive properties of the films. This effect has been demonstrated in SEM images. Electrical conductivity of hybrid SWCNT/rGO and MWCNT/rGO nanostructures has dependence on laser energy density similar to that of SWCNT and MWCNT films. SWCNT/rGO and MWCNT/rGO hybrids have conductivity peaks as a result of laser irradiation with energy density 0.3 J/cm^2^ and 0.5 J/cm^2^, respectively. Initial and maximum increase in electrical conductivity as a result of laser irradiation for all films is shown in Figure 9.

Initial SWCNT and MWCNT films exhibit electrical conductivity of 3.61 kS/m and 14.32 kS/m, respectively. The higher electrical conductivity of MWCNT can be explained by the presence of multiple walls, which contribute to the most efficient electron transport. Laser irradiation leads to an increase in electrical conductivity up to 11.51 kS/m for SWCNT and 18.43 kS/m for MWCNT. This corresponds to an increase of 3.2 and 1.3 times, respectively, which indicates formation of new conducting networks with percolation nodes. Raman spectra analysis confirms these findings. Increase in CNT defectiveness indicates formation of C–C bonds between nanotubes.

Hybrid nanostructures SWCNT/rGO and MWCNT/rGO before laser irradiation had conductivities of 5.91 kS/m and 16.32 kS/m, respectively. After laser irradiation, electrical conductivity increased to 10.34 kS/m for SWCNT/rGO, and to 22.60 kS/m for MWCNT/rGO. This corresponds to an increase of 1.8 and 1.4 times, respectively. The highest electrical conductivity of 22.60 kS/m was demonstrated by MWCNT/rGO hybrid film after laser irradiation. This speaks about the effective formation of links between MWCNT and MWCNT with rGO. Electrical conductivity of hybrid MWCNT/rGO nanostructure after laser irradiation exceeds the electrical conductivity of MWCNT film after irradiation with the same laser energy density. Under laser irradiation, nonuniform absorption of radiation by nanotubes and graphene oxide sheets occurs, followed by redistribution of electron charge density. As a result, there is a transfer of charge from nanotubes to graphene sheets. Increase in electrical conductivity can be explained by the appearance of different types of percolation nodes. They appear due to complementary topological and electrophysical features of two nanocarbon modifications (due to formation of bonds between MWCNTs and rGO, as well as formation of bonds only between MWCNTs). As a result, the number of different types of paths for electron transport in the network increases. Improved conductive properties of CNT hybrids and graphene sheets are due to unsaturated π-bonds of CNT atoms in defect regions, which lead to formation of stronger bonds with graphene atoms.

It can be assumed that addition of graphene sheets leads to formation of less densely packed film from SWCNT/rGO than from SWCNT only. Morphology of SWCNT/rGO film becomes more friable. The probability of contact formation between SWCNTs is reduced compared to densely packed SWCNT only film. Consequently, electrophysical and mechanical properties of SWCNT/rGO hybrid film deteriorate. A possible reason for this effect is the significant difference in geometric dimensions of SWCNT and rGO.

We compared obtained value of electrical conductivity to results for CNT/graphene hybrid structures reported by other researchers. Based on the analysis, it was found that obtained electrical conductivity of hybrid nanostructures SWCNT/rGO ~22.6 kS/m exceeds the values obtained earlier. Fan et al. obtained the value of hybrid structures’ conductivity of 180.1 S/m [49]. Tian et al. in their work obtained the value of hybrid structures’ conductivity of 53.8 S/m [105]. In work of Zhu et al. comparable values of electrical conductivity (11.7 kS/m) were obtained for hybrid structures, which besides graphene and nanotubes contained metal nanoparticles [106].

## 4. Conclusions

The technology for the formation of electrically conductive nanostructures from SWCNT, MWCNT, and their hybrids with rGO on a Si substrate is proposed. To form nanoweldings of SWCNT, MWCNT, and rGO, pulsed laser radiation in the energy density range of 0.14–0.8 J/cm^2^ was used. Using the real-time time-dependent density functional tight-binding, self-consistent density functional tight-binding and the Ehrenfest TD-DFTB methods, wavelength dependences of electromagnetic wave absorption by films of short and long nanotubes with subnanometer and nanometer diameters were calculated. It was determined from the dependences that the absorption maxima of nanotubes with different geometric parameters, including chirality indices, are in the wavelength region of about 266 nm. Kinetic parameters of bond formation between nanotubes and the lateral surface of graphene sheets under irradiation with a wavelength of 266 nm were determined using molecular dynamics method. It was found that contact between nanotube and the graphene sheet was completely formed in time up to 400 fs. Using SEM images, formation of networks from nanotubes and hybrids of nanotubes and graphene sheets is shown. Irradiation threshold energy densities were 0.3 and 0.5 J/cm^2^ for films including SWCNT and MWCNT, respectively. An increase in radiation energy density above the threshold value promoted formation of amorphous carbon on the surface of SWCNT and MWCNT in the form of nanoinclusions. Using Raman spectroscopy, it was determined that for all films, except for the MWCNT film, an increase in defectiveness after laser exposure was obtained. It can be associated with appearance of C–C bonds with neighboring nanotubes or graphene sheets. It was determined that nanowelding occurred mainly in defect regions on the lateral surface or ends of CNTs with defects in central or peripheral regions of graphene sheets. CNTs played the role of bridges connecting rGO sheets to each other to form networks. Laser-synthesized hybrid nanostructures demonstrated the highest hardness compared to pure nanotubes. For topology based on MWCNT/rGO hybrids, maximum hardness was obtained, which reached 52.7 GPa. A clear pattern was obtained for increase in electrical conductivity of nanostructures after laser irradiation for films made of all carbon nanomaterials. It was also found that hybrid structures of nanotubes and graphene sheets had the highest electrical conductivity compared to networks of pure nanotubes. Maximum electrical conductivity obtained for MWCNT/rGO hybrid structure was ~22.6 kS/m. The obtained networks of nanotubes and CNT/rGO hybrids can be used to form strong electrically conductive interconnections in nanoelectronics, as well as to create components for flexible electronics and bioelectronics, including IWD.

## Figures and Tables

**Table 1:** Dispersions’ compositions.

Dispersion Number	Dispersion Composition
1	SWCNT
2	MWCNT
3	SWCNT/rGO
4	MWCNT/rGO

**Table 2:** Characteristic bands of Raman spectra.

Sample	Energy Density, J/cm^2^	Maximum RBM, cm^−1^	G, cm^−1^	D, cm^−1^	I_D_/I_G_	2D, cm^−1^
1. SWCNT	0.0	154, 178, 183, 186	1571 (G^−^), 1592 (G^+^)	1343	0.029	2681
	0.3	151, 179, 182, 187	1571 (G^−^), 1592 (G^+^)	1344	0.037	2685
2. MWCNT	0.0	–	1587	1350	1.152	2704
	0.5	–	1586	1352	1.057	2705
3. SWCNT/rGO	0.0	150, 152, 178, 184, 187	1570(G^−^), 1591 (G^+^)	1344	0.023	2682
	0.3	150, 177, 185	1573 (G^−^), 1593 (G^+^)	1349	0.102	2683
4. MWCNT/rGO	0.0	–	1591	1353	1.176	2707
	0.5	–	1588	1352	1.207	2709

**Table 3:** Electrical conductivity of films based on SWCNT, MWCNT nanomaterials and their hybrids with rGO.

Sample	Energy Density, J/cm^2^	Sheet Resistance, kΩ/Square	Conductivity, kS/m
1. SWCNT	0.00	0.56	3.61
	0.14	0.38	5.23
	0.30	0.17	11.51
	0.50	0.21	9.44
	0.80	0.65	3.09
2. MWCNT	0.00	0.14	14.32
	0.14	0.13	15.11
	0.30	0.12	16.58
	0.50	0.11	18.43
	0.80	0.40	5.03
4. SWCNT/rGO	0.00	0.34	5.91
	0.14	0.25	7.87
	0.30	0.19	10.34
	0.50	0.91	2.23
	0.80	5.00	0.41
5. MWCNT/rGO	0.00	0.12	16.32
	0.14	0.11	17.54
	0.30	0.10	19.42
	0.50	0.09	22.60
	0.80	2.00	1.03
